# Experimental phasing with *SHELXC*/*D*/*E*: combining chain tracing with density modification

**DOI:** 10.1107/S0907444909038360

**Published:** 2010-03-24

**Authors:** George M. Sheldrick

**Affiliations:** a Department of Structural Chemistry, University of Göttingen, Tammannstrasse 4, D-37077 Göttingen, Germany

**Keywords:** experimental phasing of macromolecules, density modification, main-chain tracing, noncrystallographic symmetry, *SHELX*

## Abstract

The programs *SHELXC*, *SHELXD* and *SHELXE* are designed to provide simple, robust and efficient experimental phasing of macromolecules by the SAD, MAD, SIR, SIRAS and RIP methods and are particularly suitable for use in automated structure-solution pipelines. This paper gives a general account of experimental phasing using these programs and describes the extension of iterative density modification in *SHELXE* by the inclusion of automated protein main-chain tracing. This gives a good indication as to whether the structure has been solved and enables interpretable maps to be obtained from poorer starting phases. The autotracing algorithm starts with the location of possible seven-residue α-­helices and common tripeptides. After extension of these fragments in both directions, various criteria are used to decide whether to accept or reject the resulting poly-Ala traces. Noncrystallographic symmetry (NCS) is applied to the traced fragments, not to the density. Further features are the use of a ‘no-go’ map to prevent the traces from passing through heavy atoms or symmetry elements and a splicing technique to combine the best parts of traces (including those generated by NCS) that partly overlap.

## Abstract 2

Experimental phasing with *SHELXC*/*D*/*E* has been enhanced by the incorporation of main-chain tracing into the iterative density modification; this also provides a simple and effective way of exploiting noncrystallographic symmetry.

## 1. Introduction

Experimental phasing of macromolecules usually requires the presence of marker atoms such as metal atoms or sulfur in a native protein, heavy metals or halides introduced by soaking or selenium incorporated by replacing methionine with selenomethionine using a suitable expression system. In the program suite *SHELXC*/*D*/*E* (Sheldrick, 2008 ▶), every attempt has been made to reduce experimental phasing to its absolute essentials, with the aim of obtaining an interpretable electron-density map quickly and reliably rather than finding the most accurate phases. This requires some severe simplifications, for example the assumption that only one type of marker atom is present, although in practice a mixture of elements rarely causes problems. However, the approach does have the advantage of producing robust, fast and simple-to-use programs that are eminently suitable for incorporation into graphical user interfaces and automated pipelines. The programs are restricted to experimental phasing by MAD (multi-wavelength anomalous dispersion), SAD (single-wavelength anomalous dispersion), SIR (single isomorphous replacement), SIRAS (combined SAD and SIR) and RIP (phasing based on radiation-induced changes in the structure) methods. The program *SHELXC* provides a statistical analysis of the input data, estimates the marker-atom structure factors *F*_A_ and the phase shifts α and sets up the files for the other two programs. *SHELXD* (Usón & Sheldrick, 1999 ▶; Sheldrick *et al.*, 2001 ▶; Schneider & Sheldrick, 2002 ▶) is used for solving the sub­structure (*i.e.* locating the marker atoms) and *SHELXE* (Sheldrick, 2002 ▶) provides iterative phase improvement by density modification.

If the positions of the marker atoms can be located, they can be used to calculate reference phases ϕ_A_, *i.e.* the phases for the marker-atom substructure. To obtain a first approximation for the phases ϕ_T_ of the macromolecule, a phase shift α is added to these reference phases. α is estimated from the observed anomalous and/or dispersive intensity differences as outlined below,
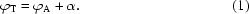
An electron-density map calculated using these approximate phases ϕ_T_ and the observed structure factors *F*_T_ may well be difficult or impossible to interpret. This is especially true for SAD phasing, where the estimates of α are restricted to 90° (when reflection *h*, *k*, *l* is significantly stronger than reflection −*h*, −*k*, −*l*) or 270° (when the opposite is true); these estimates are more reliable when the anomalous difference is large. In SAD phasing no starting phases are available for reflections corresponding to centrosymmetric projections. However, in favourable cases density modification starting from these phases, *i.e.* modifying the density iteratively so that it looks more like that expected for a macromolecule, may produce an interpretable map.

Many sophisticated density-modification schemes have been proposed, with major contributions by Peter Main, Kevin Cowtan and Tom Terwilliger, and have been incorporated into widely used programs such as *DM* (Cowtan & Main, 1998 ▶) and *RESOLVE* (Terwilliger, 2000 ▶). Possibly the first successful application of density modification, to high-resolution data for small molecules, was by Hoppe & Gassmann (1968 ▶). Effective concepts for macromolecular density modification include NCS (noncrystallographic symmetry) averaging (Main, 1967 ▶; Bricogne, 1976 ▶; Kleywegt & Read, 1997 ▶), solvent flattening (Wang, 1985 ▶), histogram matching (Zhang & Main, 1990 ▶), solvent flipping (Abrahams, 1997 ▶) and statistical approaches (Terwilliger, 2000 ▶, 2003*b* ▶; Cowtan, 2000 ▶). In this paper an alternative approach, the sphere-of-influence method (Sheldrick, 2002 ▶), will be extended by iterating it with main-chain tracing.

## 2. Discussion

### 2.1. Experimental phase information

Karle (1980 ▶) and Hendrickson *et al.* (1985 ▶) showed by algebraic analysis that given only one type of anomalously scattering atom, the diffracted intensities in a MAD experiment are given by
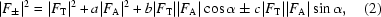
where the ‘+’ part of the ± sign refers to reflection *h*, *k*, *l* and the ‘−’ part to reflection −*h*, −*k*, −*l*. The constants *a*, *b* and *c* are functions of the complex scattering factors *f* + *f*′ + *if*′′ for the elements present: they are different for each wavelength but the same for all reflections at a given resolution for a particular wavelength. *F*_A_ is the structure factor for the marker atoms alone, ignoring the contributions from *f*′ and *f*′′, and *F*_T_ is the total structure factor for the macromolecule, including the marker atoms but ignoring the contributions from *f*′ and *f*′′. For two or more wavelengths, (2) represents an over-determined system of equations that can be solved to obtain values of |*F*_A_|, |*F*_T_| and α for each reflection. The |*F*_A_| values may then be used to solve the substructure, from which ϕ_A_ can be calculated.

For a single-wavelength (SAD) experiment, there are only two equations for the three unknowns (one for |*F*_+_|^2^ and one for |*F*_−_|^2^). If we assume that the anomalous scattering is small relative to the total scattering, the native structure factors |*F*_T_| are given to a good approximation by |*F*_T_| ≃ (|*F*_+_| + |*F*_−_|)/2. Subtraction of |*F*_−_| from |*F*_+_| in (2) and substituting for |*F*_T_| gives
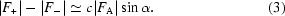
Somewhat surprisingly, these coefficients can be used in place of |*F*_A_| to locate the substructure by dual-space direct methods (Sheldrick *et al.*, 2001 ▶) using programs such as *SHELXD* that were originally developed for the *ab initio* solution of small-molecule structures. An explanation of this fortunate situation is that direct methods only employ the strongest reflections in each resolution shell and these will tend to be those with sinα close to +1 or −1, corresponding to estimated α values of 90° or 270°, respectively. Despite the use of the largest anomalous differences only, the data-to-parameter ratio for the marker-atom location will still be relatively high because of the small number of marker-atom sites. For SIR phasing, a similar analysis leads to
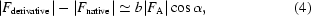
giving coefficients that can be used in place of |*F*_A_| to locate the heavy atoms and to estimated α values of 0° and 180° for the reflections with the largest isomorphous differences. In the case of SIRAS, (3) and (4) can be combined to give unbiased estimates of |*F*_A_| and α estimates in the full range 0–360°. In practice, these estimates will be less accurate than those from a MAD experiment because the native and derivative crystals will not be perfectly isomorphous. Problems of scaling in *SHELXC*/*D*/*E* are generally avoided by the use of normalized structure factors (*E* values) wherever possible, but in the case of RIP phasing some further hand-tuning is usually required (Nanao *et al.*, 2005 ▶).

### 2.2. Substructure solution

The relative |*F*_A_| (MAD or SIRAS), |*F*_A_sinα| (SAD) or |*F*_A_cosα| (SIR and RIP) calculated using *SHELXC* are con­verted to normalized structure factors (*E* values) in the dual-space direct-methods substructure-solution program *SHELXD*. *SHELXC* outputs (i) a file *.hkl containing *h*, *k*, *l*, intensity and σ(intensity) for use in density modification and possibly for later refinement with *SHELXL* (Sheldrick, 2008 ▶), (ii) a file *_fa.hkl containing *h*, *k*, *l*, *F*_A_, σ(*F*_A_) and the phase shift α for use by *SHELXD* for substructure solution and by *SHELXE* for calculating starting phases for the density modification and (iii) a file *_fa.ins containing the crystal data and instructions for running *SHELXD*. The α estimates are only required for *SHELXE*. *SHELXD* writes a *_fa.res file in *SHELX* format for the best substructure solution, which in turn is read by *SHELXE*.

It is usually more efficient to use Patterson seeding (Schneider & Sheldrick, 2002 ▶) rather than random starting atoms in the *SHELXD* substructure solution, except for high-symmetry cubic space groups in which the large number of Patterson vectors can make Patterson seeding inefficient. This seeding is performed by considering the strongest general peaks in the Patterson function as potential two-atom search fragments with a fixed vector distance between the two atoms; these vectors can be translated but not rotated. At the start of each trial, a vector is chosen pseudo-randomly from the Patterson peak list, favouring the higher peaks. A large number of random positions in the unit cell are tested for the resulting two-atom fragment; the default number is 9999 for polar space groups and 99 999 for nonpolar. The position of the two-atom fragment that gives the best Patterson superposition minimum function, based on the two atoms and all their symmetry equivalents, is used as the seed. This procedure ensures that each trail starts from a different seed that is consistent with the Patterson. The two atoms and their sym­metry equivalents are then used to generate a full-symmetry Patterson superposition minimum function; this is peak-searched to obtain further heavy-atom positions that are used to initiate the dual-space recycling. These minimum functions are calculated as the sum of the 30% weakest Patterson densities for all the vectors involved, as suggested by Nordman (1966 ▶).

A critical decision is the resolution to which the data have to be truncated for substructure solution; typically, this is determined by the resolution to which significant anomalous differences can be observed (Schneider & Sheldrick, 2002 ▶). In difficult cases up to 10 000 trials may be required per solution and the fitting of disulfides to ‘super-sulfur’ peaks can be useful in sulfur-SAD phasing (Debreczeni *et al.*, 2003 ▶). The correlation coefficient (CC) between the observed and calculated *E* values usually enables correct solutions to be identified unambiguously and the value of CC(weak), the correlation coefficient based on the reflections not used in the dual-space recycling, is also a good check. It is like a free *R* value, but is not quite independent because all of the data are used in the occupancy refinement. To allow for possible variations in occupancy, displacement parameters (*B* values) and the presence of different types of marker atoms, it has proved useful to refine the occupancies in the last two dual-space cycles. A sharp fall-off in the refined occupancy between the last true site and the first noise peak is also a useful test for a good solution, but cannot be used for halide soaks, for which a continuous range of occupancies are usually found.

### 2.3. The sphere-of-influence algorithm

The density modification in *SHELXE* does not make use of solvent flattening (which would require the generation of a solvent mask) or of histogram matching (which would require a reference histogram, *e.g.* from a related structure with the same solvent content and resolution). Instead, the sphere-of-influence algorithm (Sheldrick, 2002 ▶) is used to provide an indication as to how likely it is that each individual voxel (volume element) in the map corresponds to a true atomic site.

The variance *V* of the density on a spherical surface of radius 2.42 Å is calculated for each voxel in the map. The use of a spherical surface rather than a spherical volume was intended to save time and to add a little chemical information (2.42 Å is a typical 1,3 distance in proteins and DNA). *V* gives an indication of the probability that a voxel corresponds to a true atomic position. Voxels with low *V* are flipped (ρ′ = −ργ, where γ is usually set to 1.0). For voxels with high *V*, ρ is replaced by [ρ^4^/(ν^2^σ^2^(ρ) + ρ^2^)]^1/2^ [with ν usually 0.5 and where σ^2^(ρ) is the variance of the density ρ over the whole cell] if positive and by zero if negative. This has a similar effect to the procedure used in the *CCP*4 program *ACORN* (Yao, 2002 ▶), which however applies the same procedure to all voxels. For intermediate values of *V* a suitably weighted mixture of the two treatments is used. An empirical weighting scheme for phase recombination is used to combat model bias. It is equally likely that the substructure will possess the correct or the incorrect hand. The variance over all voxels in the asymmetric unit of the individual variances *V*, output by the pro­gram as the ‘contrast’, is a good indication of which marker-atom enantiomorph is correct; it is almost invariably higher for the correct choice, especially after 5–10 density-modification cycles. However, successful chain tracing (described below) is probably an even better indication of the correct marker-atom enantiomorph. A clear difference in the contrast between the two substructure enantiomers is a good indication that the structure has been solved. However, if the marker-atom sub­structure is centrosymmetric, for example when there are two unique heavy atoms in triclinic space groups or one unique heavy atom in monoclinic space groups, both substructure enantiomers should give similar values for the contrast and both lead to the correct structure.

A further simple and effective algorithm to improve the phases of the experimentally measured reflections is to extrapolate the data and phases to a higher resolution than was actually accessible (the free-lunch algorithm; FLA; Caliandro *et al.*, 2005 ▶; Jia-xing *et al.*, 2005 ▶); this has also been implemented in *SHELXE* (Usón *et al.*, 2007 ▶). This algorithm is effective when data have been measured to a resolution of 2.0 Å or better and can lead to improvements in the mean phase error of the measured reflections of between 5° and 30°.

### 2.4. Autotracing

A relatively fast iterative autotracing algorithm has been incorporated into the density modification in *SHELXE*. It is primarily designed to obtain a toehold in maps with very poor starting phases, *e.g.* with a mean phase error greater than 60°. The tracing proceeds as follows.

(i) Find potential α-helices in the density and try to extend them at both ends. Then find other potential tripeptides and try to extend them at both ends in the same way.
(ii) Tidy up and splice the traces as required, applying any necessary symmetry operations.
(iii) Use the traced residues to estimate phases, combine these with the initial phase information using σ_A_ weights (Read, 1986 ▶) and then restart the density modification. The refinement of one *B* value per residue provides a further opportunity to suppress wrongly traced residues.

#### 2.4.1. Searching for α-helices and other tripeptides

**Figure 1:**
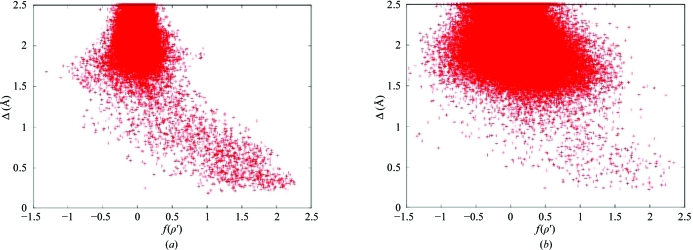
Results of the search for (*a*) seven-residue α-helices and (*b*) common tripeptides using the density obtained by *SHELXE* density modification for the 2.75 Å MAD test data for GerE (Ducros *et al.*, 2001 ▶; PDB code 1fse). Δ is defined as the average distance to the true atomic site; distances greater than 2.5 Å were replaced by 2.5 Å before calculating the average; *f*(ρ′) is defined in the text.

The chain tracing is initiated by finding seven-residue α-helices or the three most common tripeptides (Pavelcik & Pavelcikova, 2007 ▶) in the density by evaluating a weighted sum *f*(ρ′) of the modified density ρ′ at the atomic sites and also at points where, because of steric clashes with the fragments in question, no density is to be expected (‘holes’). The weights are set to the atomic numbers, except that for C^β^ (which would be absent for a glycine) the weight is set to 4 and for a ‘hole’ it is set to −2. Before performing this calculation, the density is modified so that ρ′ = ρ^1/2^ for ρ ≥ 0 and ρ′ = −|ρ|^1/2^ for ρ < 0. The starting positions for this random search are seeded using the peaks of the density, placing the peaks on the C=O bonds about 0.25 Å from the O atom. Such template searches were pioneered by Kleywegt & Jones (1997 ▶) with the program *ESSENS*. As shown in Fig. 1 ▶, the searches are appreciably more effective for α-helices than for tripeptides because of the larger number of atoms involved and also because of the smaller geometric variations.

#### 2.4.2. Extending the chains at both ends

The chain-extension algorithm looks two residues ahead of the residue currently being added and employs a simplex algorithm to find a best fit to the density at the atom centres as well as at ‘holes’ in the chain. The target function employed at each step of the chain extension is similar to that for the initial fragment search. Only torsion angles ϕ and ψ and the N—C^α^—C angles are allowed to vary, but the latter are restrained to be close to their standard values. 15 starting ϕ/ψ pairs, chosen to provide a good sampling of the populated Ramachandran regions, are employed for each peptide. Residues are added one at a time but the algorithm looks two residues ahead to decide which is the best route. The quality of each completed trace is then assessed independently before accepting it. A ‘look-ahead’ algorithm based on standard tripeptide fragments is employed in *RESOLVE* (Terwilliger, 2003*a* ▶) and *Buccaneer* (Cowtan, 2006 ▶) and a simplex algorithm is used in *Buccaneer* to refine the main chain after tracing and in *TEXTAL* (Romo *et al.*, 2006 ▶) to search for side chains. Important features of the algorithm used in *SHELXE* are the generation of a ‘no-go map’ that defines regions into which there should be no tracing, *e.g.* because of symmetry elements or existing atoms, and the efficient use of crystallographic symmetry. The trace is not restricted to a predefined volume and the splicing algorithm takes symmetry equivalents into account. It is quite common for chain tracing to be started from partially correct tripeptides in which the N- or C-terminal peptide in a tri­peptide is in fact docked into a side chain. Such chains can be recognized by the fact that they can only be extended in one direction.

#### 2.4.3. Criteria for accepting chains

The following criteria are combined into a single figure of merit for accepting traced chains.

**Figure 2:**
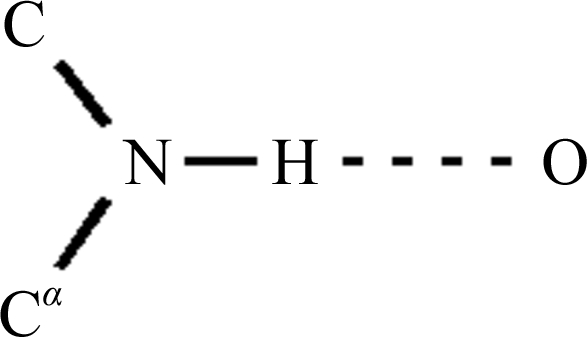
Since most main-chain amide N—H groups take part in hydrogen bonds, the density at a point found by extrapolating the N—H vector to 2.9 Å from the N atom provides an indication as to whether the amide has been positioned correctly.

(i) The modified density ρ′ should be high at the atomic sites and low at the dummy-atom positions.
(ii) The chains must be long enough (in general at least seven amino acids); longer chains are given a higher weight.
(iii) A few Ramachandran outliers can be tolerated, *e.g.* for glycines, but in general the ϕ and ψ angle pairs should lie in the well populated regions of the Ramachandran diagram.
(iv) There should be a well defined secondary structure (*i.e.* ϕ/ψ pairs should tend to be similar for consecutive residues).
(v) On average, there should be significant positive density 2.9 Å from N in the N→H direction (to a hydrogen-bond acceptor). This takes into account the fact that the large majority of main-chain NH groups in proteins take part in hydrogen bonds to oxygen or other electronegative atoms (Fig. 2 ▶).

#### 2.4.4. Splicing

**Figure 3:**
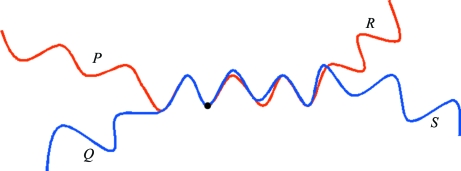
Splicing of two chains that almost coincide for part of the backbone. Firstly, the point is found at which the chains fit best, cutting each chain into two parts (*P* and *R* or *Q* and *S*). The better of *P* and *Q* (according to the figure of merit defined in the text) is spliced onto the better of *R* and *S* and the other two partial chains are discarded.

If two traces merge or cross, they are both cut into two at the point of closest contact and the best N-­terminal part is combined with the best C-terminal part (Fig. 3 ▶). Although this technique was discovered as a result of a programming error in the handling of symmetry in the no-go map, it is so effective at improving the overall quality of the map that the no-go map was redefined to allow different traces to overlap but not to allow a trace to overlap with a symmetry element, with a marker atom or with itself (which might result in a trace going round in circles). If three C^α^ atoms overlap, the chains are spliced at the middle atoms of the closest fitting groups of three C^α^ atoms; if there are no closely fitting groups of three atoms (*e.g.* because one chain does not extend far enough), overlapping pairs of atoms or single atoms are also considered. Overlapping atoms are averaged using weights that smooth out the transition from one chain to the next, but some small distortions of the main-chain geometry can still arise around the splicing points.

#### 2.4.5. Fibronectin test structure

This structure (PBD code 2cg6) was originally solved by Rudiño-Piñera *et al.* (2007 ▶), primarily by exploiting radiation damage (the UV-RIP method). At the time, this gave much better phases than long-wavelength sulfur-SAD phasing, despite the availability of a highly redundant data set collected at a wavelength of 1.77 Å on BM14 at the ESRF. These data extended to 2.0 Å resolution and the short-wavelength (0.98 Å) data to 1.5 Å resolution, but the solvent content was low (34%). Subsequent analysis showed that (as usual for sulfur-SAD) the following procedure was critical for obtaining a good sulfur substructure.

(i) Finding the right point at which to truncate the data (2.5 Å).
(ii) Using the disulfide-resolution procedure (DSUL in *SHELXD*) to locate S—S units in the peak search in each dual-space cycle.
(iii) Not being impatient! Although acceptable CC values [*e.g.* CC of 33.5% and CC(weak) of 16.2% at trial number 35] were obtained quickly, much better solutions with better peak-height distributions could be obtained by running for several thousand trials [best CC of 49.9%, CC(weak) of 30.4%].

**Figure 4:**
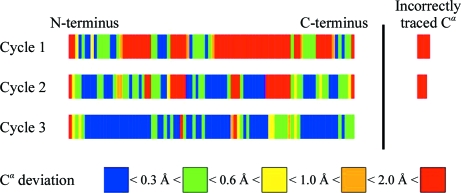
The improvement in model quality for cycles of density modification followed by autotracing for the fibronectin test structure starting from sulfur-SAD phases. The colour indicates the deviation of the C^α^ atoms from their true positions. Each row represents the protein from the N- to the C-terminus. In the first cycle, 41% was traced with C^α^ atoms within 1.0 Å, 33% within 0.5 Å and 4% incorrectly traced. After three cycles the figures were 94, 87 and 0%, respectively.

This structure illustrates the ability of the autotracing to start from a noisy sulfur-SAD map (Fig. 4 ▶). Recycling the partial (but rather accurate) traces leads to better phases and to an almost complete structure. Sulfur-SAD phasing and *SHELXE* density modification alone gave a mean phase error of 53.4° and a map correlation coefficient relative to the refined structure of 0.63. These could be improved to 42.9° and 0.70, respectively, with the FLA or to 32.3° and 0.84, respectively, using iterative autotracing. However, combining the FLA with autotracing was only slightly better than autotracing alone (31.6° and 0.86).

#### 2.4.6. GerE test structure

**Figure 5:**
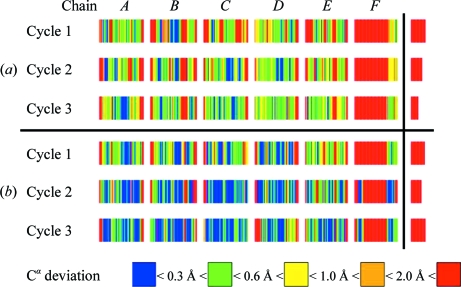
Autotracing quality for the GerE test structure using the same conventions as in Fig. 4 (*a*) for phasing using only the 2.75 Å MAD data and (*b*) after phase extension to the 2.15 Å native data.

This structure (Ducros *et al.*, 2001 ▶; PDB code 1fse) illustrates the application of *SHELXC*/*D*/*E* to a four-wavelength selenomethionine MAD experiment with data to 2.75 Å resolution. Fig. 5 ▶ shows that 70% of the C^α^ atoms are within 1.0 Å of their true position, 42% are within 0.5 Å and 3% are incorrect (more than 2.0 Å in error) when only the 2.75 Å data are used. If the phases are extended to the 2.15 Å native (sulfur) data, the figures are 78% within 1.0 Å and 69% within 0.5 Å but 6% are incorrect.

**Figure 6:**
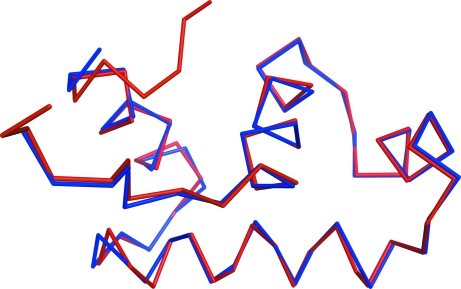
The C^α^ trace of one molecule from the MAD phasing of GerE at 2.7 Å with *SHELXE* (blue) compared with PDB entry 1fse (red). Some terminal residues are missing but otherwise the fit is good. This figure was prepared using *PyMOL* (DeLano, 2002 ▶).

Fig. 6 ▶ shows a superposition of part of the main-chain trace for the GerE structure on the structure in PDB entry 1fse.

#### 2.4.7. Including NCS in the autotracing

**Figure 7:**
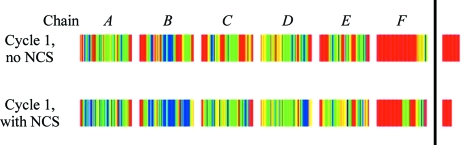
Autotracing quality after the first cycle for the GerE test structure (PDB code 1fse) using the 2.75 Å MAD data only and the same conventions as in Fig. 4 without and with NCS. Without NCS 55% of the C^α^ atoms were within 1.0 Å of their true positions and 35% were within 0.5 Å, with 6% wrongly traced. When the sixfold NCS was taken into account, the figures were 74, 49 and 3%, respectively.

NCS is normally applied to average the density of the various equivalent monomers after determining the NCS operators and molecular envelopes. In *SHELXE* the operators are derived from the heavy-atom sites but they are then applied to the traces, followed by splicing as described above, always retaining the partial traces that fit the density best. Thus, the well defined monomers help to trace the poorly defined regions, *e.g.* with higher *B* values, but there is little risk that transformed fragments from the poorly defined NCS copies will replace fragments that are already well traced. This works well for the sixfold NCS (with two marker atoms per monomer) in the 2.75 Å GerE test structure (Fig. 7 ▶), but the method still requires some fine tuning. It is fast and simple to use, in keeping with the *SHELXE* philosophy.

## 3. Conclusions

The chain-tracing algorithm and the criteria for splicing and deciding which chains to accept are the keys to the success of partial main-chain tracing in making sense of poor-quality maps. The algorithms are designed to fit part of the structure reliably rather than produce a complete backbone trace, although this has been achieved in several cases, including one previously unsolved 237-residue structure (Ni *et al.*, 2009 ▶). The idea behind the introduction of autotracing into *SHELXE* was to obtain a toehold in a noisy map, giving a partial main-chain trace and a much better map. It is important that this is fast enough to be performed while the crystal is still on the beamline. For a 2.66 GHz PC, the total *SHELXC*/*D*/*E* time for the GerE structure including one cycle of autotracing and NCS was under 3 min. When the results are sufficiently convincing, the crystal can be removed and the structure solution com­pleted later with more sophisticated programs such as *ARP*/*wARP* (Perrakis *et al.*, 1999 ▶), *RESOLVE* (Terwilliger, 2000 ▶), *Buccaneer* (Cowtan, 2006 ▶) and *Coot* (Emsley & Cowtan, 2004 ▶).

A beta test of the new autotracing version of *SHELXE* is currently being conducted by about 80 volunteers and is available on e-mail request from the author. This beta-test version also enables phases to be improved by iterative density modification and autotracing starting from a fragment obtained by molecular replacement and so can be used for MRSAD phasing (Panjikar *et al.*, 2009 ▶). It is already employed on the *Auto-Rickshaw* server at http://www.embl-hamburg.de/Auto-Rickshaw/ (Panjikar *et al.*, 2005 ▶). It is intended, as is already the case with *SHELXC* and *SHELXD*, that it will be distributed as open source when it has been fully debugged. The *SHELX* programs are also available as stand-alone binaries for common operating systems with zero dependencies on other programs or libraries.
